# The association between vaginal hygiene practices and spontaneous preterm birth: A case-control study

**DOI:** 10.1371/journal.pone.0268248

**Published:** 2022-06-30

**Authors:** Laura E. Janssen, Rubin J. T. Verduin, Christianne J. M. de Groot, Martijn A. Oudijk, Marjon A. de Boer

**Affiliations:** 1 Department of Obstetrics, Reproduction and Development Research Institute, Amsterdam UMC, Vrije Universiteit Medical Center, Amsterdam, The Netherlands; 2 Department of Obstetrics, Reproduction and Development Research Institute, Amsterdam UMC, Amsterdam Medical Center, Amsterdam, The Netherlands; University of Western Australia, AUSTRALIA

## Abstract

**Background:**

Spontaneous preterm birth (SPTB) is a major cause of neonatal morbidity and mortality worldwide and defining its risk factors is necessary to reduce its prevalence. Recent studies have pointed out that bacterial vaginosis, a disturbance in the vaginal microbiome, is associated with SPTB. It is hypothesized that vaginal hygiene practices can alter the vaginal microbiome and are therefore associated with SPTB, but there are no studies investigating this matter.

**Methods and findings:**

A case-control study was conducted between August 2018 and July 2021 in two affiliated university medical centers in Amsterdam, the Netherlands. We included a total of 79 women with a SPTB and compared them with 156 women with a term birth. Women with uterine anomalies, a history of cervical surgery or major congenital anomalies of the fetus were excluded. All participants filled in a questionnaire about vaginal washing with water, soap or gel, the use of intravaginal douches and vaginal steaming, both before and during pregnancy. Most women washed vaginally with water, 144 (61.3%) women before pregnancy and 135 (57.4%) women during pregnancy. A total of 43 (18.3%) washed with soap before and 36 (15.3%) during pregnancy. Before pregnancy, 40 (17.0%) women washed with vaginal gel and 27 (11.5%) during pregnancy. We found that the use of vaginal gel before pregnancy (aOR 2.29, 95% CI: 1.08–4.84) and even more during pregnancy, was associated with SPTB (aOR 3.45, 95% CI: 1.37–8.67). No association was found between washing with water or soap, intravaginal douching, or vaginal steaming and SPTB.

**Conclusions:**

Our findings suggest that the use of vaginal gel is associated with SPTB. Women should be informed that vaginal use of gels might not be safe.

## Introduction

Preterm birth, defined by the World Health Organization (WHO) as a delivery between 20 and 37 weeks of gestation, is a major cause of adverse neonatal outcomes worldwide [1]. Despite improvement in obstetric care, the WHO global survey indicates that both non-spontaneous and spontaneous preterm birth (SPTB) are a rising problem. The incidence of SPTB in Europe is estimated to be 5.5 to 11.1 percent [2]. Due to the significant risk of adverse outcomes for preterm born children, identifying risk factors for SPTB is of great importance [3]. Currently, several risk factors have been identified by epidemiological studies, such as a history of SPTB, high or low maternal age, black race and low education or low socioeconomic status, cervical surgery, multiple pregnancy, tobacco use and low maternal weight [4].

Multiple studies pointed out that bacterial vaginosis (BV) is also associated with SPTB [5–9]. BV is an imbalance of the normal vaginal flora, with overgrowth of anaerobic bacteria and a reduction of the Lactobacillus species [10]. A meta-analysis including over 30.000 women from 32 studies showed that BV approximately doubled the risk of preterm delivery in asymptomatic patients; OR 2.16, 95% CI: 1.56–3.00 [11]. The longitudinal study of Brotman et al. indicated that intravaginal douching, a practice of intravaginal cleaning that includes insertion of a liquid solution in the vagina through a tube, was practiced more often by women who had BV [12]. Most commercial douche products consist primarily of fragrance, acetic acid, and water, some also contain surfactants, such as Octoxynol-9 or Cetylpyridinium Chloride [13]. Surfactant detergents can disrupt lipid membranes and thus have antimicrobial and viricidal activities. Additionally, these detergents may wash away antibacterial agents or disturb cell membranes, causing irritation to mucosal surfaces, which in turn can increase susceptibility to genital tract infections [14].

Because of the association between BV and SPTB, it was hypothesized that intravaginal douching is associated with SPTB. This hypothesis is supported by several studies [15–17]. However, most of these studies included a substantial amount of African American women, who already are at higher risk for SPTB and might use intravaginal douches more often, which lowers the generalizability of these results.

Vaginal steaming with herbal preparations is of common use in Indonesia, Thailand, and South-Africa [18, 19]. European women are thought to practice vaginal hygiene more often by vaginal washing with water, soap or by using vaginal (over the counter) gels. There are no studies investigating the association between these vaginal hygiene practices and SPTB, which is of great interest since its use can be altered easily by educating women early in pregnancy.

In conclusion, there remains a lack of knowledge about the incidence of vaginal hygiene practices during pregnancy and the associated risk of SPTB. Therefore, in this study we investigated the incidence of vaginal hygiene practices in women with a SPTB and compared them to women with a term birth.

## Materials and methods

We performed a case-control study at two locations of the Amsterdam University Medical Center (Amsterdam UMC) in the Netherlands, between August 2018 and July 2021. Cases were women with a SPTB during the study period which was defined as delivery between 22+0 and 36+6 weeks of gestation that started with spontaneous contractions or spontaneous rupture of membranes. Controls were women that delivered after 37 weeks of gestation in the same study period. Exclusion criteria for both cases and controls were uterine anomalies, a history of cervical surgery (conisation or radical surgery), multiple pregnancy, major congenital anomalies of the fetus, age under 18 years at the time of pregnancy or the inability to read Dutch or English.

### Procedure and measurements

Eligible women were informed about the study within 24 hours after delivery. After signing informed consent, all participants were sent an E-mail with a link to an online self-reported questionnaire. We created a non-validated questionnaire addressing vaginal hygiene, see S1 Data. Women were asked about the use of the following vaginal practices, both before and during pregnancy: vaginal washing with water, soap, or (over the counter) gels, the use of intravaginal douches or vaginal steaming and what kind of herbs were used. The questionnaire also addressed the frequency of use before and during pregnancy, which was divided into four categories: daily, more than once a week, weekly, or sporadic. If used during pregnancy, timing of last use was categorized in during last week, last month and more than a month before delivery. The questionnaire also addressed ethnicity, level of education, sexual intercourse frequency, and altered vaginal discharge during pregnancy. Clinically relevant data, such as gravidity, parity, gestational age at delivery, age, and obstetric history was abstracted from the medical records. This study was approved by the medical ethical committee of the VU Medical Center and Amsterdam Medical Center, study approval number 2018.298.

### Sample size calculation

Although we investigated multiple practices, sample size could only be calculated using intravaginal douching, as only this variable is investigated in previous literature. Approximately 14% of white women in the United States use vaginal douches regularly [20]. We therefore expected the prevalence of intravaginal douching in Europe to be approximately 10%. We hypothesized that the prevalence of intravaginal douching was 25% in women who had SPTB, since research shows that 25% of the women with BV use vaginal douches [20]. To achieve 80% power to detect a difference between the group proportions of 15%, the study needed 78 cases and 155 controls. The test statistic used to calculate the sample sizes, is the two-sided Z test with pooled variance. The significance level of the test was targeted at 0.05.

### Statistical analysis

Patient characteristics were examined using Chi-square test, Fisher’s exact test, independent samples t-test or Mann-Whitney U-test. All calculations to obtain corresponding p-values were two-sided. The characteristics for continuous variables were presented as mean and standard deviation (SD), variables with a skewed distribution as median and interquartile range [IQR]. Categorical variables were presented as percentages of numbers for corresponding group. Differences between cases and controls regarding vaginal hygiene practices were assessed using Chi-squared test. To investigate the association between hygiene practices and SPTB, the Odds ratio’s (OR) and their corresponding 95% confidence interval (CI) were calculated using logistic regression analysis. A multivariable analysis was performed to adjust for potential confounders using hierarchical backward elimination, including covariates that were moderately associated with preterm birth (p<0.1). We performed a subgroup analysis based upon of the severity of SPTB: extreme (22+0–27+6 weeks of gestation), very (28+0–31+6 weeks of gestation) and moderate preterm (32+0–36+6 weeks of gestation). Sub classification was based on the WHO definitions of preterm birth [21]. P-values <0.05 were considered statistically significant. All analyses were performed using the statistical package SPSS V.28.

## Results

**Fig 1:**
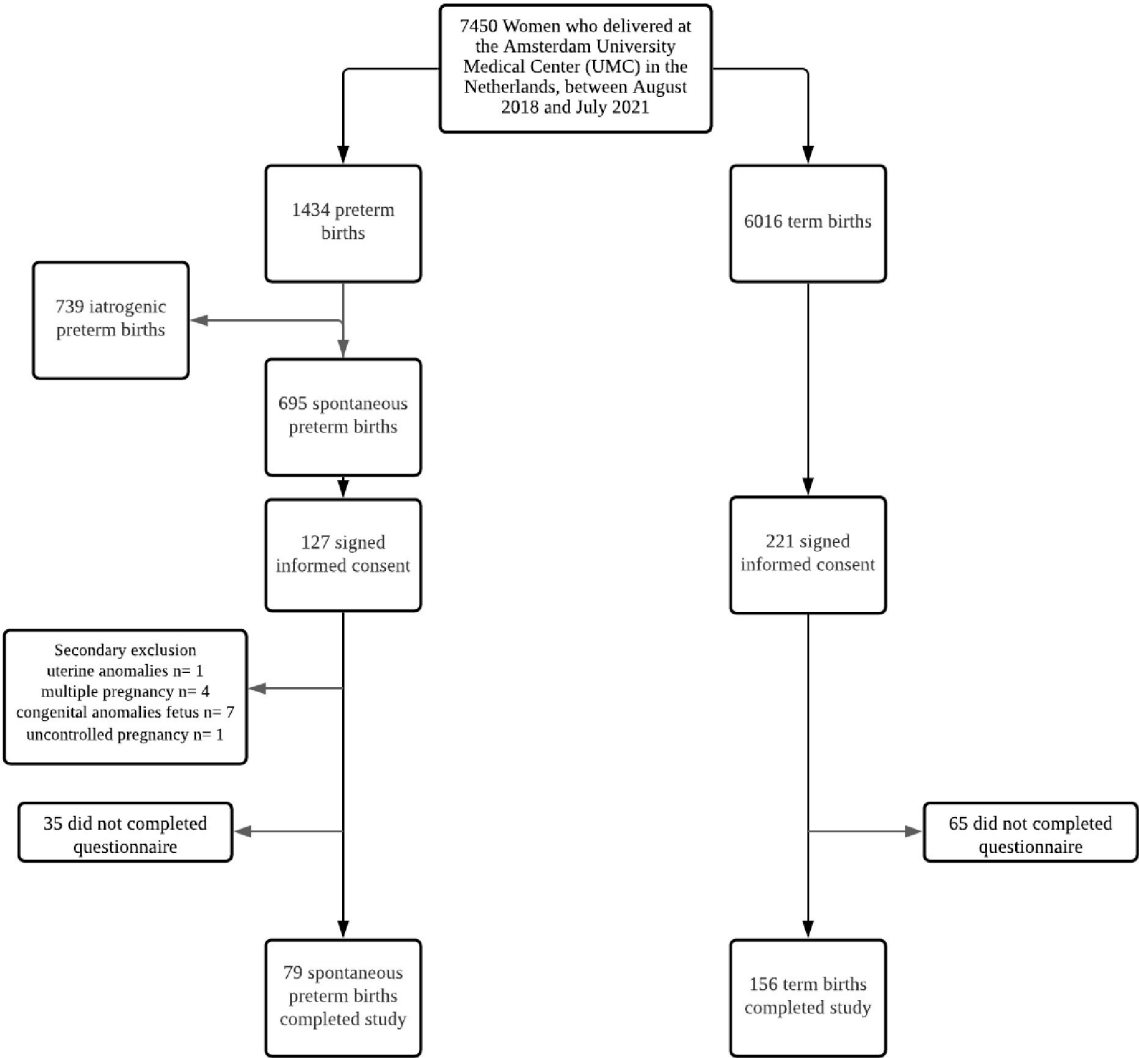
Flowchart of the recruitment of the study population.

The flowchart of the study population is shown in Fig 1. A total of 7450 women gave birth at the Amsterdam UMC hospital between August 2018 and July 2021 of which 695 women had a SPTB. A total of 127 women with SPTB signed informed consent of which 13 were secondary excluded. Eventually 79 cases filled in the complete questionnaire. A total of 221 with a term birth signed informed consent and a total of 156 completed the questionnaire and no controls were excluded.

Baseline characteristics of the study population are shown in Table 1. Women with SPTB were more often primiparous and more often had a previous preterm delivery. Other baseline characteristics were comparable between the groups.

**Table 1:** Characteristics of the study population.

	SPTB N = 79	Term birth N = 156	P-value
Maternal age	31.82 ± 4.68	32.97 ± 4.84	.083
Prepregnancy BMI	23.50 [20.6–26.2]	23.54 [21.6–27.5]	.280
Caucasian^a^	55 (69.6)	98 (62.8)	.302
Level of education^b^			.266
Low	0 (0.0)	6 (3.8)	
Intermediate	23 (29.1)	36 (23.1)	
High	55 (69.6)	111 (71.2)	
Unknown	1 (1.3)	3 (1.9)	
Smoking during pregnancy	1 (1.3)	3 (1.9)	1.0
Parity^c^			
1	58 (73.4)	81 (51.9)	.002
2	15 (19.0)	49 (31.4)	.045
≥3	6 (7.6)	26 (16.7)	.070
Previous preterm delivery^d^	10 (47.6)	17 (22.7)	.025
History of miscarriage^e^	13 (43.3)	56 (55.4)	.243
History of curettage^e^	5 (14.7)	13 (11.3)	.561
History of LLETZ	4 (5.1)	3 (1.9)	.229

Values are mean ± SD or n (%) or median [IQR]

BMI, body mass index; LLETZ, Large Loop Excision of the Transformation Zone; SPTB, spontaneous preterm birth

^a.^ Country of birth of participant and the minimum of one parent or both parents despite participant in Europe, Western Asia, Central Asia, North Africa, and the Horn of Africa

^b.^ Low: primary education, lower general secondary education; Intermediate: high school, intermediate vocational education; High: pre-university, higher vocational education, and university

^c.^ Parity after index pregnancy

^d.^ Excludes primipara women

^e.^ Excludes primigravid women

The use of vaginal hygiene practices is shown in Table 2. Most women washed vaginally with water, which was reported by a total of 144 (61.3%) women before pregnancy and 135 (57.4%) women during pregnancy. A total of 43 (18.3%) washed with soap before pregnancy and 36 (15.3%) during pregnancy. Before pregnancy, 40 (17.0%) women washed with vaginal gel which accounted for 20 (25.3%) cases and 20 (12.8%) controls (p = .016). During pregnancy, vaginal gel use was also more often used by cases (n = 15, 19.0% vs. n = 12, 7.7%, p = .010). A total of 5 (2.1%) women used intravaginal douches and a total of 2 (0.9%) women practiced vaginal steaming when they were not pregnant. During pregnancy, none practiced intravaginal douching and vaginal steaming was practiced by 1 (0.4%) woman. Being Caucasian and sexual intercourse was not associated with vaginal hygiene practices, results not shown.

**Table 2:** Vaginal hygiene practices for cases and controls.

	SPTB N = 79	Term birth N = 156	P-value
Use before pregnancy			
Washing with water	50 (63.3)	94 (60.3)	.652
Washing with soap	13 (16.5)	30 (19.2)	.722
Washing with gel	20 (25.3)	20 (12.8)	.016
Intravaginal douching	2 (2.5)	3 (1.9)	1.00
Steaming	0 (0.0)	2 (1.3)	.552
No hygienic measures	19 (24.1)	39 (25.0)	1.00
Use during pregnancy			
Washing with water	46 (58.2)	89 (57.1)	.863
Washing with soap	10 (12.7)	26 (16.7)	.420
Washing with gel	15 (19.0)	12 (7.7)	.010
Intravaginal douching	0 (0.0)	0 (0.0)	NA
Steaming	1 (1.3)	0 (0.0)	.336
No hygienic measures	21 (26.6)	49 (31.4)	.445

SPTB, spontaneous preterm birth; NA, not applicable

Values are n (%)

**Fig 2:**
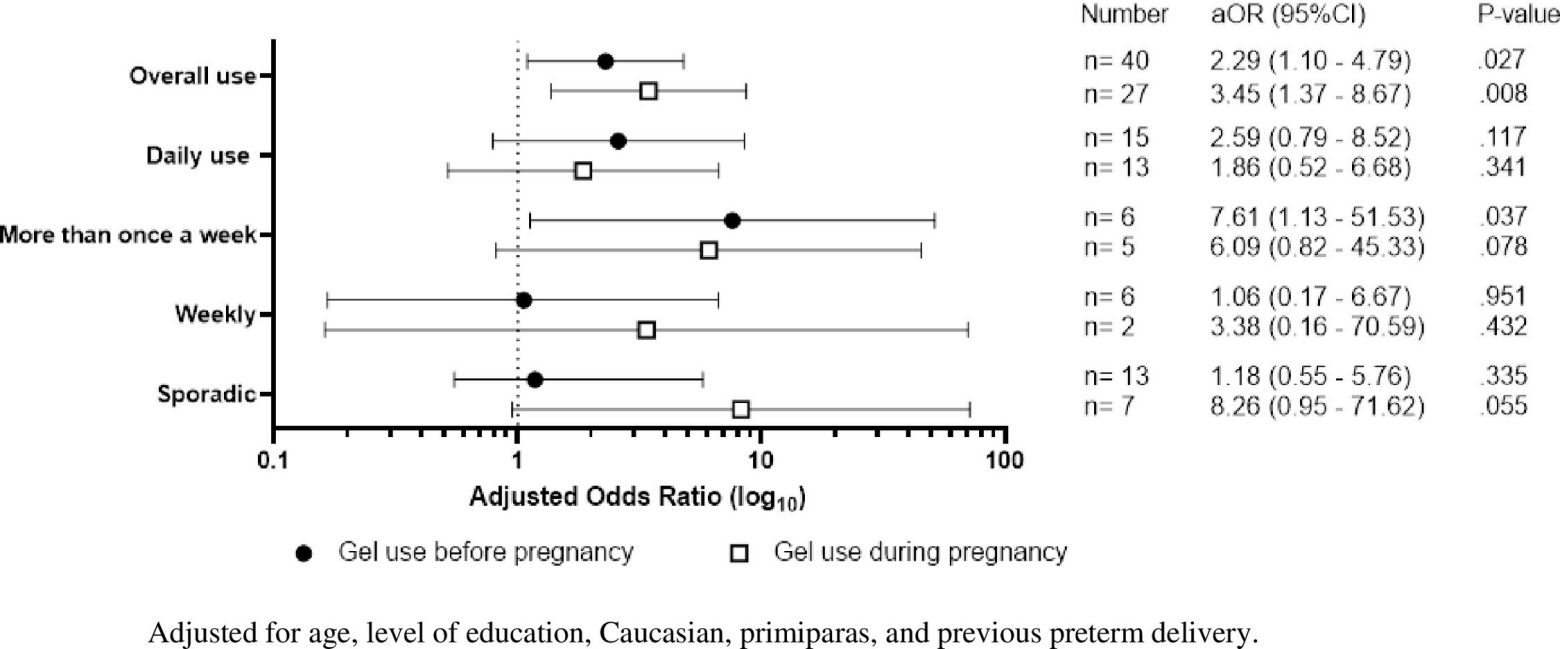
Adjusted odds ratio (AOR) and 95% confidence intervals (CI): Frequency of vaginal gel use and the risk of spontaneous preterm birth, versus no vaginal gel use.

Vaginal gel use before pregnancy was associated with SPTB; OR 2.31, 95% CI: 1.16–4.60, p = .016. In the multivariable analyses adjusted for age, level of education, Caucasian, primiparas, and previous preterm delivery, the association between gel use before pregnancy and SPTB remained significant; aOR 2.29, 95% CI: 1.10–4.79, p = .027. Gel use during pregnancy was also associated with SPTB, also after adjustment for previously mentioned confounders; aOR 3.45, 95% CI: 1.37–8.67, p = .008. Women who washed vaginally with gel more than once a week before pregnancy had the highest risk of SPTB; aOR 7.61, 95% CI: 1.13–51.53, p = .037. Results are shown in Fig 2. Timing of vaginal gel use during pregnancy was not associated with the risk of SPTB, results not shown.

Subgroup analysis based upon the severity of SPTB, showed that gel use before pregnancy mainly increased the risk of SPTB between 28+0 and 31+6 weeks of gestation; aOR 2.65, 95% CI: 1.04–6.75, p = .042. Vaginal gel use during pregnancy also showed a significant association with SPTB between 28+0 and 31+6 weeks of gestation; aOR 3.70, 95% CI: 1.17–11.74, p = 0.03. A stronger association was found for vaginal gel use during pregnancy and SPTB between 32+0 and 36+6 weeks of gestation; aOR 5.01, 95% CI: 1.56–16.13, p = < .001. Results are shown in Table 3.

**Table 3:** Subgroup analysis based upon the severity of spontaneous preterm birth compared with term birth. Adjusted odds ratios and 95% confidence intervals.

	Extreme preterm (GA 22+0–27+6 weeks) n = 13	Very preterm (GA 28+0–31+6 weeks) n = 40	Moderate preterm (GA 32+0–36+6 weeks) n = 26
Used before pregnancy			
Washing with water	2.87 (0.65–12.65)	1.12 (0.50–2.53)	0.72 (0.29–1.79)
Washing with soap	2.44 (0.61–9.67)	0.53 (0.17–1.72)	0.47 (0.13–1.66)
Washing with gel	2.27 (0.48–10.78)	2.65 (1.04–6.75)*	2.22 (0.77–6.38)
Used during pregnancy			
Washing with water	3.10 (0.72–13.40)	0.85 (0.39–1.87)	0.67 (0.27–1.64)
Washing with soap	1.07 (0.24–4.87)	0.28 (0.06–1.29)	0.74 (0.22–2.42)
Washing with gel	1.29 (0.12–14.09)	3.70 (1.17–11.74)*	5.01 (1.56–16.13)*

* P-value <0.05

Adjusted for age, level of education, Caucasian, primiparas, and previous preterm delivery.

## Discussion

To our knowledge, this case-control study is the first to investigate the association between multiple vaginal hygiene practices and SPTB and showed a significant association. We found that vaginal washing with gel, both before and during pregnancy was significantly associated with an increased risk of SPTB, in particular SPTB between 28+0 and 31+6 weeks of gestation. No association between other hygiene practices, such as washing with water or soap, intravaginal douching and steaming and SPTB were found.

There is no existing literature on the use of vaginal gel and obstetric outcomes, a Canadian cross-sectional survey from 2018 found that non-pregnant women using vaginal gel sanitizers had a greater risk for reporting a yeast infection or for reporting BV [22]. As for other vaginal practices, we found that vaginal washing with soap or water, both before and during pregnancy, was not associated with SPTB. There are a few studies that investigated the association between these vaginal hygiene practices and BV and sexual transmitted infections, which are known to be potential contributors to SPTB [11, 23]. Joesoef et al. showed that the vaginal use of water did not increase the susceptibility for sexual transmitted infections in pregnant women [24]. A study from Rajamanoharan et al, who examined the use of genital cleaning agents and BV found that women who washed the vulvar area or the vaginal area with soap, did not have an increased risk for developing BV [25]. A more recent study from Sabo et al. showed that both vaginal washing with soap or water was not associated with several vaginal bacteria related to vaginal dysbiosis and BV [26]. These results are in line with our data, suggesting that use of both water and soap do not influence the vaginal health environment, leading to adverse outcomes. For water it might be explained by the fact that it is a non-toxic, pH-neutral, substance. However, for soap, it is generally believed that the high pH levels in soaps will disrupt the degree of acidity in the vaginal environment, commercial brands selling vaginal gels use this believe to promote their products, as they insinuate that their product would avoid the disruption of the acidic environment that soap potentially causes [27].

Other studies reported a significant association between intravaginal douching and SPTB [15–17]. Unfortunately, we were not able to support or contradict this finding due to low frequencies of intravaginal douching in our study. The low number of women who douched in our study may be explained by a lowering douching frequency over time because of previous research that reported adverse outcomes [15–17]. Another reason is that we included a small number of women with an African American descent, whereas previous studies enrolled a substantial amount of African American women, who are known to have a higher incidence of intravaginal douching [15–17, 28].

We included a relatively big cohort with a sample size of 235 women from two large academic centers in the Netherlands with different demographic backgrounds which was representative for the general Dutch population. An additional strength of our study was the detailed information collected on vaginal hygiene behavior as we provided a comprehensive questionnaire, which addressed multiple hygiene practices with their timing and frequency. Furthermore, we were able to adjust for possible confounding factors.

Some limitations also need to be addressed. Ideally, a correlation between BV or vaginal infections and the use of the different vaginal hygiene practices was made. However, this was not possible in our study because vaginal swabs are not standard of care in our centers. We tried to address this important question by asking for alterations in vaginal discharge since abnormal vaginal discharge may be a clinical symptom of dysbiosis of the vaginal microbiome. This analysis did not reach significance. As we included patients in the first week after birth, results were sensitive for recall bias. Further, it is possible that women with SPTB were afraid to honestly report their vaginal hygiene behavior through believes that these practices may accounted for the adverse outcome of SPTB. We were not fully able to adjust our results for socioeconomic status as we did not collect data about this matter. This covariate had the potential to be a confounding factor, due to the relation with both SPTB and the use of vaginal douches [29]. Not adjusting our results for this covariate could risk the internal validity by a potential overestimation of the observed association between vaginal hygiene practices and SPTB. We experienced low inclusion percentages, although this is common in this kind of research where inclusion is asked in the first week after birth, it has the potential for selection bias.

We used a non-validated questionnaire, although questions were carefully selected, they could be misinterpreted by the participants. For example, our questionnaire did not clearly differentiated between internal and external vaginal washing. Therefore, it is not fully clear whether women might have interpreted vaginal use as the application of agents in the vaginal area, or intravaginal. Approximately 60% of the women reported to wash vaginally with water both before and during pregnancy, therefore we assume that at least part of the participants interpreted vaginal washing as external vulvar cleaning.

Our results show that the use of vaginal gels before and during pregnancy might not be safe. Commercial brands that sell vaginal gels, claim that gel use supports or even restores the vaginal pH, since these gels have a pH value similar to that of the vagina. In contrast, some brands recommend their product should be solely applied to the vulvar area, and even suggests that vaginal use may be harmful to the internal vaginal environment [27].

In our study, the significant association between vaginal gel use and SPTB, was mainly present in the very preterm birth between 28+0 and 31+6 weeks of gestation. This finding supports a theory that vaginal gel use promotes infections, since infections are the leading cause for preterm birth for the gestational age period between 22 and 32 weeks [30, 31]. The potential pathophysiologic pathway towards infections by vaginal gel use may be caused by an alteration in the microbiome, leading to BV, increasing the susceptibility for vaginal pathogens to gain access to the upper genital tract or upper part of the uterus. However, if BV may only be a marker, instead of a mediator, for a potentially weakened vaginal immune barrier, this would explain why previous attempts to reduce preterm birth by treating BV failed [32]. Another possibility for increased susceptibility for infections might include dysfunction of the vaginal epithelial cells, which normally produce protective and anti-inflammatory mediators [33]. Vaginal gels in the Netherlands contain, among other ingredients, Propylene Glycol, Lactic Acid and Glycerin, which has been shown to irritate and damage the vaginal epithelial cells in both humans and animals [34, 35]. At last, it might be possible that intensive vaginal cleansing products such as gels and douches cause a loss of the multiple layers of dead or dying cells in in the vaginal environment, which are believed to protect against infection, resulting in a higher vulnerability [36]. Since we can only be speculative about the pathophysiologic pathway behind gel use that leads to SPTB, we recommend future studies to investigate whether vaginal hygiene practices lead to alteration of the vaginal microbiome and whether these alterations are associated with SPTB.

## Conclusions

In conclusion, the use of vaginal gels, increased the risk of SPTB when used before and during pregnancy. We advise health-care professionals to discourage the use of vaginal gels in pregnant women and women attempting to conceive.

## Supporting information

S1 Data Questionnaire of the study. (PDF)

S2 Data Database of the study. (XLSX)
