# Supplementary material for: The association between vaginal hygiene practices and spontaneous preterm birth: A case-control study
Source: PLoS One. 2022 Jun 30;17(6):e0268248. doi: 10.1371/journal.pone.0268248 (PMC9246112; doi:10.1371/journal.pone.0268248)
Supplement: S1 Data — (PDF) [file pone.0268248.s001.pdf]

ALPHA-STUDY
The questionnaire

A study about vaginal hygiene and preterm birth

Personal data

1. What is the date of your delivery? …. - …. - ……..

2. What is your country of birth?

   …………………………………………………………

3. What is the country of birth of your father?

   …………………………………………………………

4. What is the country of birth of your mother?

   …………………………………………………………

5. What etnicity do you regard yourself?
   Tick the most appropriate box, choose only one option. Then go to question 6.

   ☐ Dutch
   ☐ Turkish
   ☐ Moroccan
   ☐ Surinamese
   ☐ Dutch Antillian
   ☐ Other, namely: …………………………………………………..

6. What is your highest level of education?
   Tick the most appropriate box, choose only one option. Then go to the questionnaire.

   ☐ Elementary school
   ☐ Pre-vocational secondary education (VMBO)
   ☐ Tertiairy education (HAVO)
   ☐ Pre-university education (VWO, Gymnasium or Atheneum)
   ☐ Further vocational education (MBO)
   ☐ Higher vocational education (HBO)
   ☐ Academic higher education (WO)
   ☐ Other, namely: …………………………………………………..
   ☐ I don’t want to answer this question

Questionnaire

1. Did you use one of the vaginal hygiene practices below, <u>when you were not pregnant</u>?
   Tick the most appropriate box(es), you can select more than one answer. Then go to question 2. When your answer is 'no': go to question 3.

   ☐ Vaginal steaming with herbs. If yes, which herbs did you use?
   I used the following herbs for vaginal steaming: …………………………………………….........
   ☐ Using intravaginal douches like Multi-Gyn
   ☐ Vaginal washing with water
   ☐ Vaginal washing with soap
   ☐ Vaginal washing with gels like Lactacyd or Balance Activ
   ☐ Other, namely: …………………………………………………..
   ☐ No, I did not use these practices when I wasn't pregnant
   ☐ I don't want to answer this question

2. How often did you use the practice(s), <u>when you were not pregnant</u>?
   Tick the most appropriate box for every practice you chose in question 1. Then go to question 3.

	Daily	Multiple times a week	Weekly	Monthly	Sporadic
Vaginal steaming					
Intravaginal douching					
Washing with soap					
Washing with water					
Washing with gel					
Other: ………………………….					

3. Did you use one of the vaginal hygiene practices below, <u>during this pregnancy</u>?
   Tick the most appropriate box(es), you can select more than one answer. Then go to question 4. When your answer is 'no': go to question 6.

   ☐ Vaginal steaming with herbs. If yes, which herbs did you use?
   I used the following herbs for vaginal steaming: …………………………………………….........
   ☐ Using intravaginal douches like Multi-Gyn
   ☐ Vaginal washing with water
   ☐ Vaginal washing with soap
   ☐ Vaginal washing with gels like Lactacyd or Balance Activ
   ☐ Other, namely: …………………………………………………..
   ☐ No, I did not use these practices when I wasn't pregnant
   ☐ I don't want to answer this question

4. How often did you use the practice(s), during this pregnancy?
Tick the most appropriate box for every practice you chose in question 3. Then go to question 5.

	Daily	Multiple times a week	Weekly	Monthly	Sporadic
Vaginal steaming					
Intravaginal douching					
Washing with soap					
Washing with water					
Washing with gel					
Other: ……………………….					

5. When did you use the practice(s) for the last time during this pregnancy?
Tick the most appropriate box, choose only one option. Then go to question 6.

☐ During the last week of my pregnancy
☐ During the last month of my pregnancy
☐ Before the last month of my pregnancy

6. Did you notice increased vaginal discharge during this pregnancy?
Tick the most appropriate box, choose only one option. Then go to question 7.

☐ No I didn't notice this
☐ Yes, but only slightly more than before my pregnancy
☐ Yes, I clearly noticed increased vaginal discharge during my pregnancy
☐ I don't want to answer this question

7. Have you been diagnosed with a vaginal infection by a doctor or midwife, during this pregnancy?
Tick the most appropriate box(es), you can select more than one answer. Then go to question 8.
When your answer is 'no': go to question 9.

☐ Yes, I had bacterial vaginosis or gardnerella
☐ Yes, I had a yeast infection (e.g. Candida)
☐ Yes, I had a sexually transmitted disease (e.g. Chlamydia or Gonorrhea)
☐ No, I haven't been diagnosed with a vaginal infection during my pregnancy
☐ I don't want to answer this question

8. Did you receive a treatment for your vaginal infection during your pregnancy?
Tick the most appropriate box, you can select more than one answer. Then go to question 9.

☐ Yes, I received oral treatment
☐ Yes, I received vaginal treatment
☐ No, I didn't receive a treatment during my pregnancy
☐ I don't want to answer this question

9. Did you have sexual intercourse during this pregnancy and if yes, how often?
Tick the most appropriate box, choose only one option. After this you reached the end of the questionnaire.

☐ No sexual intercourse during this pregnancy
☐ Less than once a month
☐ Less than once a week
☐ 1-2 times a week
☐ 3-4 times a week
☐ 5 or more times a week
☐ Other, namely: ……………………………………………………..
☐ I don't want to answer this question

This is the end of the questionnaire. Thank you for your participation.
